# Many purported pseudogenes in bacterial genomes are *bona fide* genes

**DOI:** 10.1186/s12864-024-10137-0

**Published:** 2024-04-15

**Authors:** Nicholas P. Cooley, Erik S. Wright

**Affiliations:** 1 Department of Biomedical Informatics, University of Pittsburgh, Pittsburgh, PA USA; 2 Center for Evolutionary Biology and Medicine, Pittsburgh, PA USA

**Keywords:** Genomics, Error correction, Misassembly

## Abstract

**Background:**

Microbial genomes are largely comprised of protein coding sequences, yet some genomes contain many pseudogenes caused by frameshifts or internal stop codons. These pseudogenes are believed to result from gene degradation during evolution but could also be technical artifacts of genome sequencing or assembly.

**Results:**

Using a combination of observational and experimental data, we show that many putative pseudogenes are attributable to errors that are incorporated into genomes during assembly. Within 126,564 publicly available genomes, we observed that nearly identical genomes often substantially differed in pseudogene counts. Causal inference implicated assembler, sequencing platform, and coverage as likely causative factors. Reassembly of genomes from raw reads confirmed that each variable affects the number of putative pseudogenes in an assembly. Furthermore, simulated sequencing reads corroborated our observations that the quality and quantity of raw data can significantly impact the number of pseudogenes in an assembler dependent fashion. The number of unexpected pseudogenes due to internal stops was highly correlated (R^2^ = 0.96) with average nucleotide identity to the ground truth genome, implying relative pseudogene counts can be used as a proxy for overall assembly correctness. Applying our method to assemblies in RefSeq resulted in rejection of 3.6% of assemblies due to significantly elevated pseudogene counts. Reassembly from real reads obtained from high coverage genomes showed considerable variability in spurious pseudogenes beyond that observed with simulated reads, reinforcing the finding that high coverage is necessary to mitigate assembly errors.

**Conclusions:**

Collectively, these results demonstrate that many pseudogenes in microbial genome assemblies are actually genes. Our results suggest that high read coverage is required for correct assembly and indicate an inflated number of pseudogenes due to internal stops is indicative of poor overall assembly quality.

**Supplementary Information:**

The online version contains supplementary material available at 10.1186/s12864-024-10137-0.

## Background

Microbial genomes often harbor pseudogenes that are remnants of previously functional coding sequences. Pseudogenization is a step toward gene removal and may result from the absence of selective pressures that maintain a functioning gene product [1]. As such, pseudogenes sometimes comprise a substantial portion of the genome of obligate symbionts that no longer require functions provided by the host [2]. Bacteria also use pseudogenization as a means of regulation, wherein a subset of the population contains a frameshift that disables a protein’s function [3]. The number of pseudogenes can vary enormously in genomes from the same species, suggesting pseudogenization is a major mode of evolution. However, pseudogenes can also result from errors in genome sequencing or assembly, and it is presently unclear how many pseudogenes are spurious pseudogenes that are technical artifacts of genome sequencing and assembly.

Most pseudogenes are defined by only a single mutation [4], suggesting they have not had time to degrade further, are adaptive variants [5, 6], or resulted from technical artifacts introduced during the sequencing and assembly process. Pseudogenes offer a potential means to interrogate assembly fidelity, because many errors would result in the false appearance of gene degradation. For example, short insertions and deletions (indels) that are not a multiple of three in length result in frameshifts that may create a defective or truncated protein product [7]. Similarly, incorrect base calls may appear as nonsense mutations that split a real gene into separate fragments. The presence of an elevated number of pseudogenes therefore implies a reduced (or reversed) selection pressure on the maintenance of genes [8] and/or errors in the genome assembly. Disentangling these two possibilities is difficult because there is no clear signature of genuine versus spurious pseudogene mutations. Nevertheless, the number of pseudogenes has important implications for our understanding of genome evolution, and studying pseudogenes may reveal best practices for acquiring genomes with high fidelity.

During routine experiments with a strain of *E. coli* in our laboratory, Sanger sequencing revealed that several (6 of 10 selected) pseudogenes were actually errors in a public genome sequence (Fig. S1). Therefore, we set out to more broadly determine the extent and causes of false pseudogenes in publicly available genomes. To this end, we developed approaches for discerning real from spurious pseudogenes, which we show can result from assembly of low coverage or low quality sequencing data. Our analysis was facilitated by the NCBI’s Prokaryotic Genome Annotation Pipeline (PGAP) that identifies pseudogenes through homology searches [9]. We focused on pseudogenes arising from frameshifts and internal stops because they permit us to differentiate indels from base miscalls, respectively. Using a combination of observational and experimental data, we were able to identify multiple causes of elevated pseudogene counts in publicly available genomes.

## Results

### Indirect evidence that some pseudogenes are artifactual

**Fig. 1:**
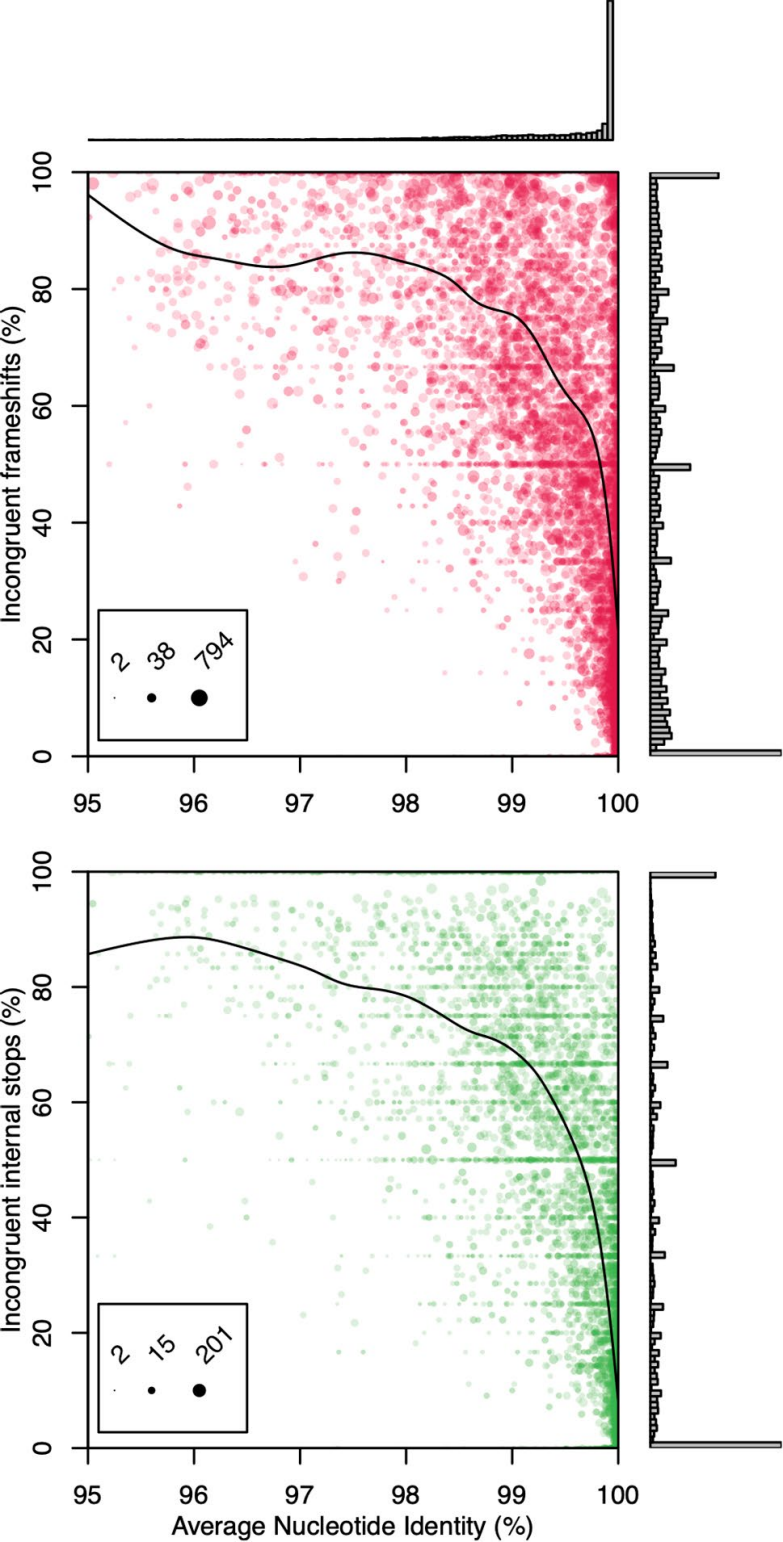
Pseudogene discrepancies do not converge to zero near 100% ANI. The percentages of frameshifts (top) and internal stops (bottom) that are incongruent (pseudogene/gene) pairs are shown for 10,362 genomes and their nearest neighbor in RefSeq. Up to 100 genomes were randomly selected from each genus in RefSeq and coding sequence pairs were identified with Clusterize at ≥ 90% similarity. Each point represents the nearest genome by average nucleotide identity (ANI) scaled to the number of matched pseudogenes. Spline fits (curves) show that disagreement in whether a coding sequence is a gene or pseudogene does not converge to zero as two genomes approach identity. Inset legends show the scaling of point sizes based on the total number of congruent or incongruent pseudogene pairs shared by both assemblies

We rationalized that as two genomes approach 100% average nucleotide identity (ANI) their number of pseudogenes should also approach equality. To investigate this, we randomly selected 100 genome assemblies from all 121 bacterial genera in RefSeq with at least 100 assemblies. For every assembly, we determined its nearest neighbor within the same genus according to ANI. We then clustered all coding sequences in each pair of genomes with at least 90% nucleotide similarity. We defined the fraction of incongruent pseudogenes as the count of pseudogene/gene pairs divided by the total number of pseudogene/pseudogene and pseudogene/gene pairs, where the pair partners each originated from the two different assemblies. Figure 1 shows that as ANI approaches 100%, the fraction of incongruent pseudogenes between genome pairs decreases. Yet, there were many genome pairs having a nearest neighbor with very high ANI (~100%) and a high fraction of incongruent pseudogenes. We estimated that pairs of nearly identical genomes differed on average by 8.2% of pseudogenes attributed to internal stops and 21.6% of pseudogenes attributed to frameshifts. This result implies there is a source of stochasticity in the number of pseudogenes, but it does not reveal the source.

### Technical variables are observationally implicated in the number of pseudogenes

**Fig. 2:**
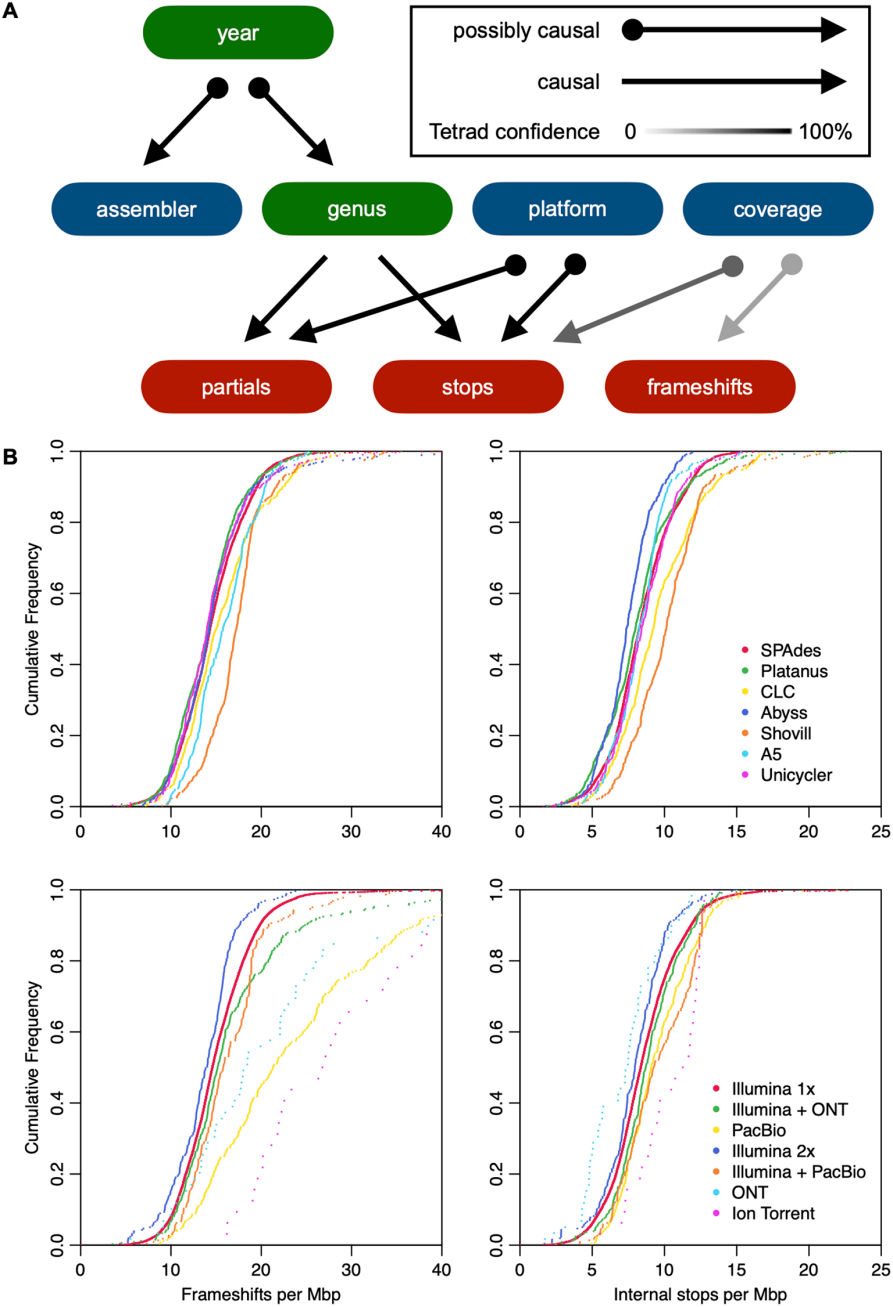
Potential causes of variability in pseudogene counts. (**A**) Reported (blue) and automatic (green) metadata may affect the number of putative pseudogenes (red) in a genome assembly. Arrows represent causal connections predicted by Tetrad between metadata variables for available RefSeq genomes. Sequencing platform and read coverage were predicted to have a potential causal influence over observed pseudogene counts. (**B**) Cumulative distributions of average pseudogene density per assembly identified by PGAP in publicly available *E. coli* assemblies. Some reported assemblers and sequencing platforms were associated with unusual numbers of frameshifts (left) and internal stops (right). However, this observational dataset cannot do more than suggest hypotheses as to the causes of variability in pseudogene counts. Inset legends are ordered from most to least frequently observed assembler (top row; *n* = 7480 total) or sequence platform (bottom row; *n* = 10,170 total)

It is notoriously difficult to discern causation from observational data because of confounding factors. In an attempt to address this difficulty, we used the fast causal inference algorithm in the causal discovery program Tetrad [10] to infer variables that causally influence the number of pseudogenes observed in 126,564 RefSeq assemblies with associated metadata. Tetrad predicted that the sequencing platform (100% confidence) and read coverage (62% confidence) were causal predictors of the number of pseudogenes due to internal stops, or there existed an unmeasured confounding factor (Fig. 2A). Only read coverage was predicted to potentially causally influence the number of pseudogenes due to frameshifts, albeit with low confidence (37%). To further investigate these factors, we focused on *E. coli* since it was the genus with the most assemblies. Genome assemblies derived from Illumina reads generally had the fewest pseudogenes attributed to internal stops or frameshifts (Fig. 2B). There was also a difference in the number of pseudogenes due to reported assembler. Statistically significant variation (Table S1) in the number of pseudogenes held for the four most commonly observed species in RefSeq (Fig. S2). Nevertheless, these results are based on observational data and controlled experiments are required to validate potential causal factors underlying elevated pseudogene counts.

### Assembler choice causally influences the number of pseudogenes

**Fig. 3:**
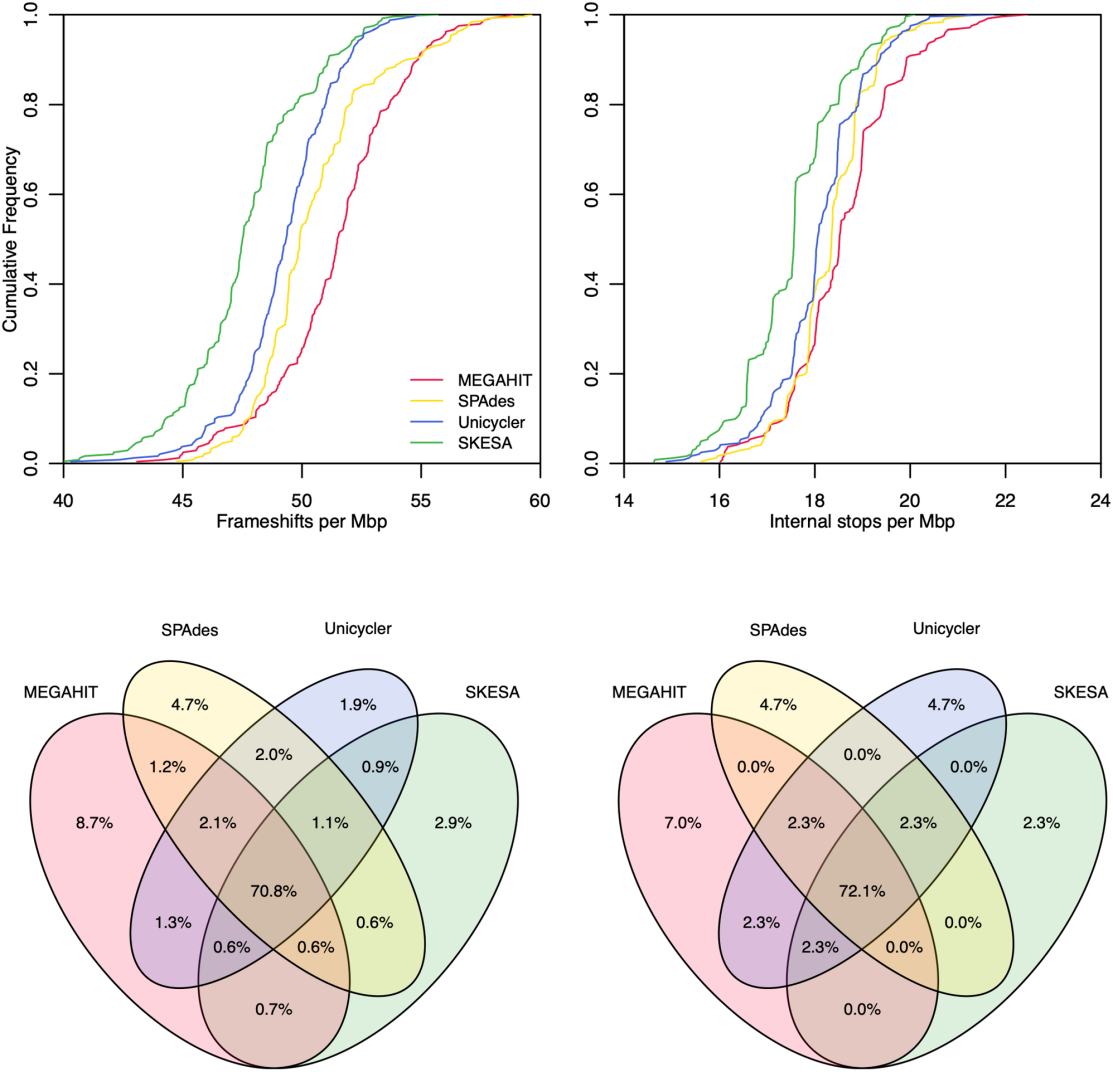
Choice of assembler causally influences pseudogene counts. Cumulative distributions for frameshifts per Mbp (top left) and internal stops per Mbp (top right) for reassembled *N. gonorrhoeae* genomes with available Illumina reads (*n* = 242). Pseudogenes were matched across the four reassemblies of each genome at ≥ 90% similarity with Clusterize. Venn Diagrams show the overlap in frameshifts (bottom left) and internal stops (bottom right) for the reassembled genomes. Overall, only about 70% of pseudogenes were shared by all four assemblers

We next sought to investigate the role of variables that were observationally implicated in the number of pseudogenes. To this end, we reassembled genomes belonging to *Neisseria gonorrhoeae*, a species known to use phase variation to regulate gene expression in a population by means of frameshifting [11] and likely to include many legitimate pseudogenes. We used four different assemblers to reassemble Illumina reads in the Short Read Archive (SRA) associated with *N. gonorrhoeae* genomes and annotated the resulting genomes with PGAP. We used Clusterize [12] to match coding features across assemblies of the same genome. The assembler had a substantial influence over the number of pseudogenes attributed to internal stops or frameshifts (Fig. 3). Only about 70% of pseudogenes were shared by assemblies from all four assemblers. MEGAHIT [13] created the most pseudogenes and also the most pseudogenes not found by any other assembler, while SKESA [14] generated the fewest pseudogenes. SPAdes [15] and Unicycler [16] had the most overlap, which was expected given that Unicycler relies on SPAdes for assembly. However, there was no way for us to determine the true number of pseudogenes and, therefore, this controlled experiment only permitted us to validate a causal effect of assembler on pseudogene counts.

### Sequencing platform and run causally influence pseudogene counts

**Fig. 4:**
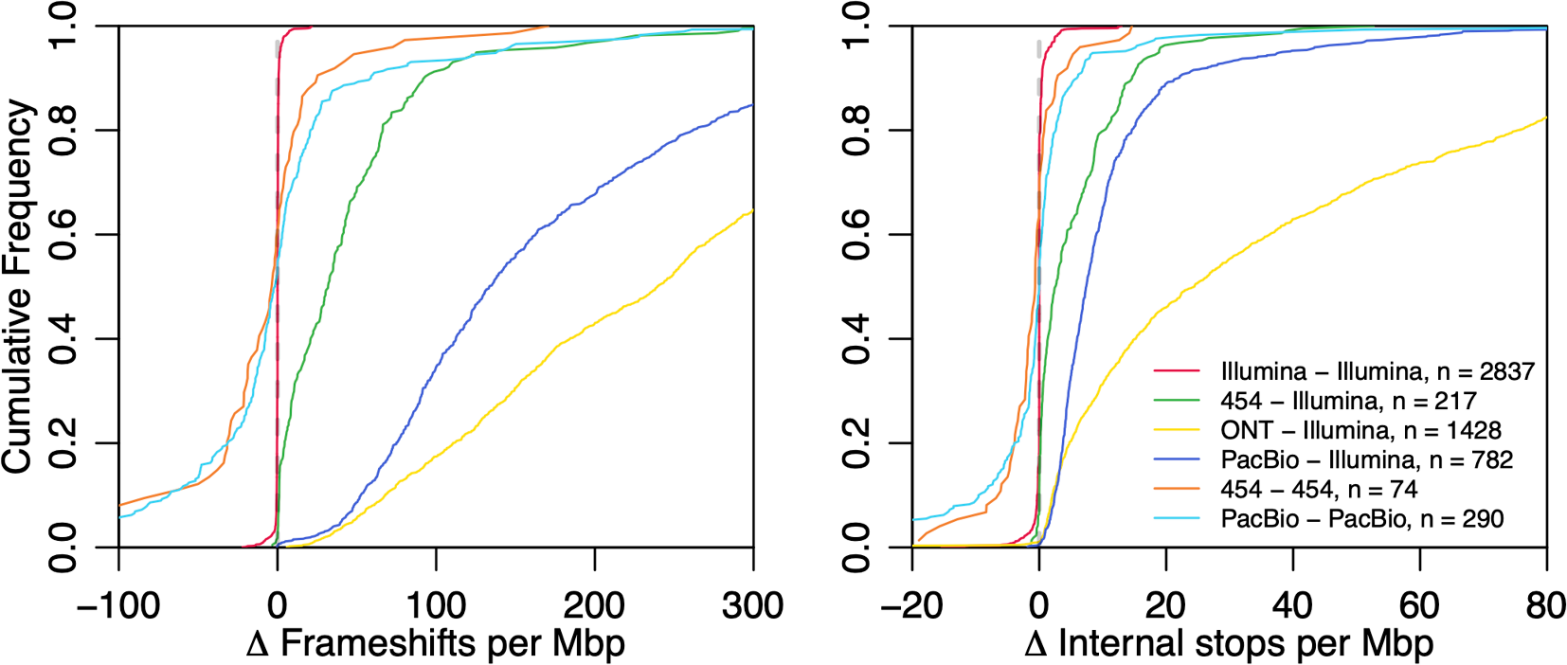
Replicate sequencing runs often differ in pseudogene counts. Cumulative distributions of the differences between frameshift (left) and internal stop (right) counts per Mbp for BioSamples with at least two unique sequencing runs deposited in the SRA. While Illumina sequencing runs of the same genome were highly repeatable, other sequencing technologies sometimes resulted in dramatic variability in pseudogene counts

Having explored the influence of assembler, we next investigated the role of sequencing platform and run on pseudogene counts. To do so, we on the fact that some genomes are sequenced more than once on the same or different sequencing platforms and the resulting reads are separately deposited in the SRA under the same BioSample accession. This enabled us to control for the effect of organism on the number of pseudogenes. We reassembled all reads corresponding to BioSamples with two or more SRA accessions (i.e., runs) belonging to the same genome using Unicycler, because it can assemble both short and long reads. Assemblies of Illumina reads from independent SRA accessions showed very little variation in pseudogene counts (Fig. 4). In contrast, assemblies from all other sequencing platforms showed greater variation between runs, particularly for pseudogenes attributed to frameshifts. Also, assemblies from Illumina reads typically resulted in fewer pseudogenes than other sequencing platforms, especially relative to assemblies exclusively derived from long reads (Fig. 4). These results demonstrate that sequencing platform has a substantial influence over the number of pseudogenes, but do not definitively reveal which sequencing platform results in the most correct pseudogenes since the correct number is unknown.

### Simulated reads reveal the direct effect of variables on pseudogenes

Having established that assembler and sequencing technology causally influence the number of pseudogenes, we next sought to quantify the influence of variables on the generation of spurious pseudogenes. To this end, we used the ART read simulator [17] to generate reads of known coverage and quality from an existing genome. We decided to use the *E. coli* K-12 genome (GCF_000005845.2) because it has been extensively studied and is well-annotated. Replicate batches of simulated Illumina reads were generated at varying qualities and coverages broadly encompassing the typical ranges observed in the SRA. We used three different error profiles representing the HiSeq X, HiSeq 2500, and MiSeq Illumina platforms. Single-end and paired-end reads were then assembled with four different assemblers. The resulting assemblies were annotated with PGAP and coding features were mapped to the original *E. coli* assembly using Clusterize [12]. We also used PBSIM3 [18] to simulate long reads at 20-fold coverage and standard quality to use with Unicycler in creating hybrid assemblies. Finally, we fit a binomial model to the normalized number of spurious pseudogenes per assembly as a function of coverage and quality (Fig. S3).

**Fig. 5:**
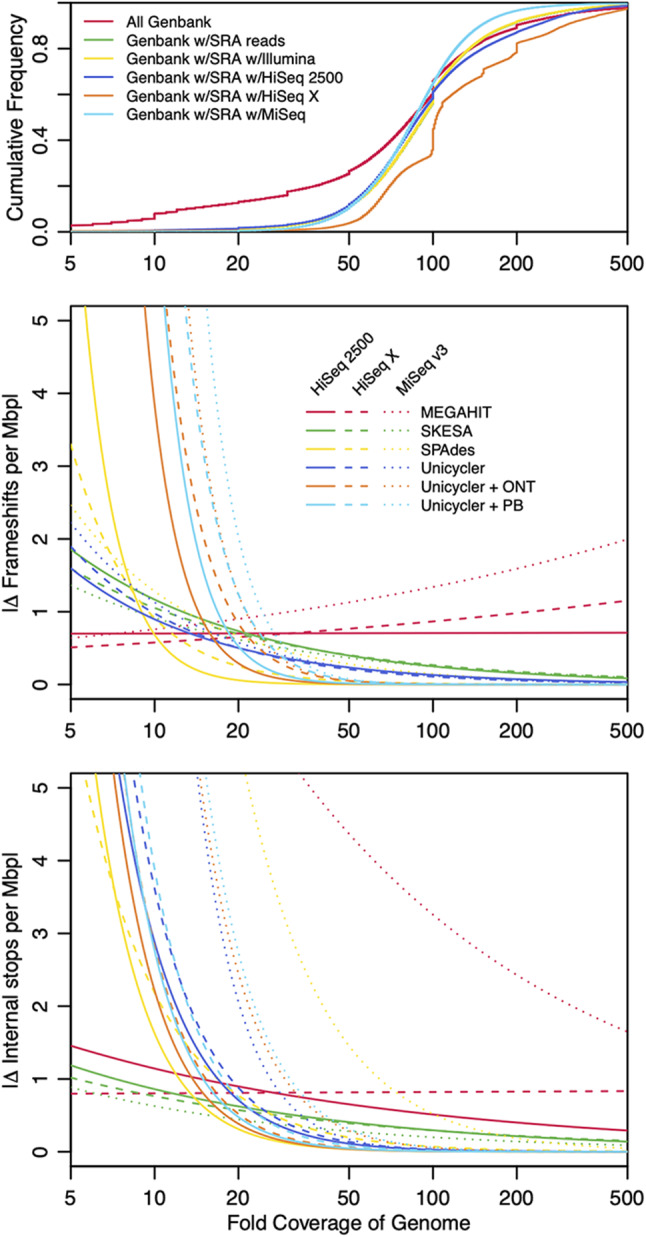
Modeled pseudogene deviation as a function of read coverage. Curves show the binomial model fits of the absolute difference in frameshifts (center) and internal stops (bottom) per Mbp relative to the representative *E. coli* genome for assemblies generated from simulated Illumina reads with an average Q-score of 35. Pseudogene differences generally increase rapidly with decreasing read coverage. However, the rate of change depends markedly on the combination of sequencing platform and assembler. Reported fold-coverage for 1,661,482 prokaryotic GenBank assemblies (top) imply that many publicly available assemblies were generated from reads with insufficient coverage to achieve low pseudogene error

As shown in Fig. 5, no spurious frameshifts or internal stops were created when assembling paired-end reads at ≥ 200-fold coverage or hybrid assemblies (i.e., long and short reads) at ≥ 50-fold short read coverage. However, assembling long reads with short reads generated many more spurious pseudogenes at low coverage (< 20) than short reads alone, presumably due to over-reliance on lower quality long reads. Low quality reads primarily increased the number of internal stops rather than frameshifts (Fig. S4). Single-end reads required 1000-fold coverage to largely eliminate spurious pseudogenes (Fig. S5). Sequencing platform affected the number of pseudogenes in a repeatable way across assemblers, with the MiSeq platform resulting in more spurious pseudogenes. Choice of assembler influenced the number of pseudogenes in a quality and coverage dependent manner, with some assemblers performing better at low coverage and worse at high coverage relative to others.

When placed in context of reported bacterial assembly coverages in GenBank (Fig. 5), these results imply the existence of many publicly available bacterial genomes containing pseudogenes that are *bona fide* genes. In fact, 8% of GenBank assemblies with associated Illumina reads have 10-fold or less reported coverage, wherein we would expect at least one spurious pseudogene per million base pairs (Mbp) even under favorable simulation conditions. Only 67.2% of GenBank assemblies have associated SRA reads, and 98.9% of those are from the Illumina platform. Assemblies linked to Illumina SRA reads tended to have higher coverage than GenBank overall, and our simulation results suggest these assemblies are possibly higher quality.

### An elevated number of pseudogenes is indicative of problematic assemblies

There is considerable need to identify and filter problematic assemblies given the anticipated abundance of errors in public genome repositories. Genome completion is typically quantified by the number of contigs or N50, although completeness is not the same as correctness. Recently, it was suggested to use deviation from the expected distribution of coding sequence lengths to identify anomalous assemblies [19]. We found that the number of contigs, N50, and deviation in coding sequence length all were weakly correlated or uncorrelated with ANI (Fig. S6) between the reassembled and original *E. coli* assemblies. In contrast, absolute difference in the number of pseudogenes attributed to internal stops was highly correlated (R^2^ = 0.961) with overall ANI. These results suggest the relative number of internal stops between a genome and a highly similar neighbor is a good proxy for overall assembly error. Notably, even though some reassembled genomes had no pseudogene differences from the original *E. coli* genome, no reassembled genome perfectly recapitulated the original. This implies the relative number of internal stops is a reasonable proxy for overall assembly quality, but its resolution is insufficient to distinguish two almost identical genomes.

Given that the number of pseudogenes attributed to internal stops was a good proxy for ANI, we considered how this number could be used to filter anomalous assemblies. As shown in Fig. 1, the deviation in incongruent internal stops trended sharply downward as ANI approached 100%. Therefore, for each assembly with a nearest neighbor having at least 99.9% ANI, we filtered the assembly if its number of pseudogenes was statistically significantly greater than its neighbor. This approach effectively accounts for the role of taxa on the number and type of pseudogenes. Specifically, we performed a binomial test of whether each assembly’s number of pseudogenes due to internal stops was greater than the 99th percentile expected for a genome with its number of coding sequences but its nearest neighbor’s rate of pseudogenes due to internal stops. Applying this test resulted in the rejection of 3.6% of assemblies in RefSeq having a nearest neighbor with at least 99.9% ANI.

### Real reads show greater variance in spurious pseudogenes than simulated reads

Our results using simulated reads implicated low coverage assemblies as a major source of spurious pseudogenes. However, these results could not explain the existence of high coverage assemblies with elevated pseudogene counts. We reasoned that taxonomy or other factors uncaptured by our simulations may contribute to spurious pseudogenes. We asked whether very high coverage assemblies could be used to explore the effect of these variables. Doing so requires assuming that very high coverage assemblies provide a baseline for comparison to lower coverage genomes. To this end, we randomly subsampled replicate sets of reads at multiple coverages and compared them to the assembly generated from reads at ~1000-fold coverage. This allowed us to fit a binomial model to the difference in pseudogenes as a function of coverage, as in our simulated experiments (Fig. 5). From this model, we predicted two values: the expected difference in pseudogene density at 50-fold coverage and the coverage required to achieve only a single pseudogene difference per Mbp.

**Fig. 6:**
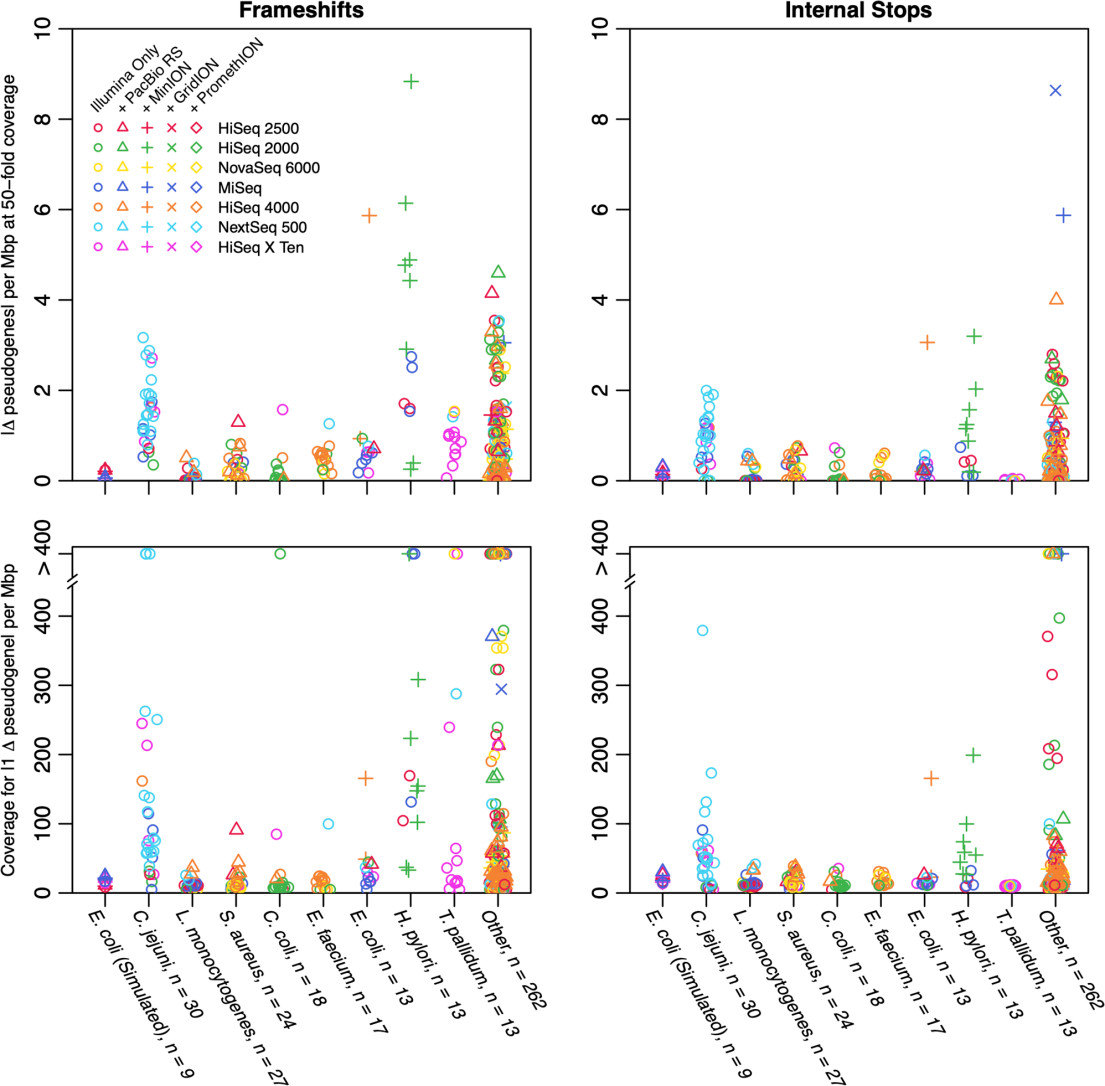
Fitted deviations in pseudogene differences for high-coverage assemblies. Reads corresponding to very high coverage (≥ 1000-fold) assemblies were reassembled with Unicycler to explore the effect of taxonomy and coverage on the generation of spurious pseudogenes. Reassemblies differed considerably in the density of spurious pseudogenes at 50-fold coverage (top row) and the coverage required to reach only a single pseudogene difference per Mbp (bottom row). This result implies considerable variability in spurious pseudogene counts in real data beyond that observed for simulated reads, which are shown by the leftmost points. Only species with at least 13 independent read sets (n) are shown, with the remainder merged into the “Other” category (rightmost points)

As shown in Fig. 6, real reads resulted in more variation than simulated reads, both within and across taxa. While 50-fold coverage was largely sufficient with simulated *E. coli* paired-end reads, much higher coverages of real reads were required to mitigate spurious pseudogenes in some cases. This result held across different read sources, including hybrid (short and long) read assemblies. The high degree of variability within each species implies the existence of additional factors beyond those tested here, which may include strain-level differences, DNA extraction protocols, sequencing machines [20], or interactions among error sources [21]. Hence, it is difficult to establish a single threshold for sufficient coverage or use coverage as a surrogate for assembly error, but higher coverage assemblies should generally be expected to contain fewer pseudogenes than lower coverage assemblies.

## Discussion

Pseudogenes have been extensively studied for their roles in evolution [22–24]. To our knowledge, this study is the first to examine pseudogenes as artifacts of the sequencing and assembly process. We showed that spurious pseudogenes arise from low quality or low coverage reads at a rate that is dependent on the sequencing platform and method of assembly. Our results imply that the relative number of pseudogenes can be used as a proxy for overall assembly quality and indicate high coverage is critical for minimizing spurious pseudogenes. However, in no instance did we go full circle from a known genome back to the same genome via simulated sequencing followed by reassembly. This result is consistent with recent work showing that it remains difficult to assemble an error free bacterial genome [25–27]. Genomes should therefore be expected to contain errors beyond those captured by filtering on the number of pseudogenes.

The main strength of our study is the variety of approaches we used to investigate spurious pseudogenes. However, our study is not without limitations. First, we relied exclusively on PGAP for annotation of pseudogenes. Different annotation programs find alternative numbers of pseudogenes in the same genome [28, 29], indicating pseudogene detection is somewhat program dependent. Second, in some cases we focused on select taxonomic groups, and these findings may have been taxon-specific. Third, it is impossible to know the true number of pseudogenes in a genome, so we relied partly on simulated reads to validate our observational conclusions. Simulations necessitate many assumptions and cannot holistically account for all variability in real sequencing data. We would expect true reads to encompass other error modes not captured in simulations [20, 30, 31]. Therefore, we suspect our simulation results represent a low estimate of the number of spurious pseudogenes, as confirmed by the reassembly of high-coverage genomes. Fourth, we focused on pseudogenes due to internal stops and frameshifts, although PGAP also reports pseudogenes due to unexpectedly truncated coding sequences. We chose to ignore truncations since these pseudogenes can result from incomplete assemblies. Notwithstanding these limitations, we anticipate that our main results are generalizable because of their repeated observation across multiple approaches.

We did not assess the presence of pseudogenes in metagenome-assembled genomes (MAGs), which is a type of assembly recently added to RefSeq. There are many programs specific to metagenome assembly [32] and error detection or correction [33–35], which is well-known to be a difficult task [36, 37]. Similarly, assemblies exclusively from long reads are likely to become more common as their error rates improve [38, 39], although we did not include solely long read assemblies in our simulations due to their high simulated error rates [40]. The increasing number of deposited genomes assembled exclusively from long reads further intensifies the need to control for assembly error in publicly available genomes. We anticipate the relative number of pseudogenes being a useful proxy for overall assembly quality as genome repositories continue to diversify. Excluding genomes assembled from low coverage reads and reads exclusively from error prone sequencing platforms is an important first step in limiting errors. Our results reinforce calls to carefully follow potential errors through analysis pipelines as errors continue to accrue in public datasets [41].

This study highlights the importance of depositing raw sequencing reads and clearly connecting them to existing assemblies, which unfortunately was not the case for many genomes currently available. We believe that access to raw reads is critical for transparency and confidence in the quality of data present in genome repositories. Raw reads permit reassembly with different assemblers and allow the verification of support for specific mutations. Studies of pseudogenes would be wise to verify the existence of frameshifts and internal stops using raw sequencing data and, ideally, resequencing. At the time of this study, less than half of prokaryotic assemblies in RefSeq had associated reads deposited in the SRA, and in some cases there were insufficient metadata to explain which of multiple sequencing runs were used to produce an assembly. Enforcing the collection of this information is currently a missed opportunity for public genome repositories such as GenBank.

## Conclusions

In this study, we found that many bacterial pseudogenes are, in fact, *bona fide* genes and care should be taken when drawing conclusions from pseudogenes. The number of spurious pseudogenes due to internal stops or frameshifts was influenced by the sequencing technology, read coverage, read quality, and assembler. We found that the number of pseudogenes relative to other highly similar genomes can serve as a proxy for differences in overall assembly quality and potentially be used to filter anomalous assemblies. The results of this study imply that there are many errors incorporated in publicly available genomes and the accumulation of assembly errors may result in over-estimation of genetic diversity across organisms. Compensating for these errors necessitates that depositing raw sequencing reads becomes commonplace and public genome repositories should continue to work toward this goal. Achieving this goal may enable repositories such as RefSeq to reassemble genomes from their raw reads to provide standardized assemblies. Additionally, we highly recommend repositories consider using pseudogene counts as another way to flag potentially anomalous assemblies. Certainly many pseudogenes are not artifactual and pseudogenes will continue to play an important role in microbial evolution. Nevertheless, our results strongly caution against lending too much credence to pseudogenes found in public assemblies without alternative evidence for their veracity.

## Methods

### Acquisition of genome assemblies and associated metadata

Edirect (v19.1) tools were used to acquire assembly metadata from RefSeq release 212 and GenBank release 255. Database queries are provided in the GitHub repository associated with this study. To exclude assemblies with previously known issues, the results were filtered to assemblies that were not flagged by NCBI as partial, anomalous, and failing taxonomy check. Metadata included the FtpPath_RefSeq, BioSampleAccn, AssemblyStatus, SubmissionDate, Taxid, SpeciesName, ContigN50, ScaffoldN50, Coverage, and total_length. General feature format (gff) files were parsed to extract feature locations and pseudogene types. Each assembly’s assembly_stats file was parsed to extract reported assemblers and sequencing technology. Metadata, including Run, LibraryStrategy, LibrarySelection, LibrarySource, Platform, Model, and BioSample, was collected for each BioSample record associated with an assembly’s SRA identifier. The RefSeq dataset contained 226,398 assemblies with 111,431 SRA runs, and the GenBank dataset contained 1,661,482 assemblies with 1,130,514 SRA runs. Assemblies and annotations were downloaded using the FtpPath_RefSeq metadata, while runs were downloaded as reads using the SRAtoolkit (v3.0.5) with the ‘split-3’, ‘skip-technical’, ‘read-filter pass’, and ‘clip’ arguments.

### Comparison of paired assemblies

Bacterial genera (*n* = 121) with at least 100 members, including Candidatus genera, were used to quantify the relationship between genome similarity and pseudogene counts. Each genus was randomly subset to 100 distinct assemblies for computing pairwise ANI with the ANI calculator [42]. Related coding sequences were identified with the *Clusterize* function in the R [43] package DECIPHER (v2.28.0) [12] at a nucleotide similarity threshold of at least 90%. Clusters containing more than two sequences, suggesting the existence of paralogous gene copies, were excluded from analysis. The percentage of incongruent pairs (Fig. 1) was then calculated as the number of clustered pairs containing a pseudogene (from one genome) and a gene (from the other genome) normalized by the number of pairs containing two pseudogenes (i.e., congruent) or a pseudogene and a gene (i.e., incongruent). Spline fits in Fig. 1 were made with the *smooth.spline* function in R while weighting each point by the number of congruent plus incongruent pseudogene pairs.

### Causal modeling of pseudogene counts using assembly metadata

RefSeq metadata was encoded for causal modeling by removing ‘Candidatus’ from genus names, as well as integer encoding assembly status, assembler, technology, and genus. Additional columns were added to the dataset for submission year, N50 normalized by total assembly length, and pseudogenes per Mbp due to internal stops, frameshifts, or incompleteness (i.e., partial). Assemblies with missing or aberrant (e.g., zero coverage) metadata were excluded from analysis. Genera, submission years, assemblers, and technologies with fewer than 1,000 entries were excluded. Causal edges among variables under the submitter’s control were forbidden (e.g., assembler cannot cause genus). Causal inference was conducted with Tetrad (v1.3.0) using the fast causal inference algorithm [10].

### Reassembly of *Neisseria gonorrhoeae* genomes with different assemblers

To elucidate the effect of assembler on pseudogenes, we focused on the species *N. gonorrhoeae* owing to its use of phase variation, abundance of BioSamples, and relatively unskewed distribution of pseudogene counts. BioSamples with associated paired-end Illumina reads corresponding to RefSeq assemblies with at least 100-fold reported coverage were selected from the SRA metadata. Adapter sequences were removed with fastp and quality trimmed with the *TrimDNA* function in DECIPHER [12]. Assembly was performed with SKESA (v2.5.1), SPAdes (v3.15.5) with ‘--isolate’ and ‘--cov-cutoff auto’ arguments, Unicycler (v0.5.0), and MEGAHIT (v1.2.9). Successful assemblies were annotated with PGAP and pseudogene types were extracted from the resulting gff files. Pseudogenes were tabulated by type and assembler before creating a Venn diagram using the VennDiagram package in R.

### Contrasting independent sequencing runs of the same sample

A total of 11,577 BioSample records had at least two associated sequencing runs that could be used to compare run-to-run variability. Assembly was performed using Unicycler defaults with a minimum contig length of 200 nucleotides. Any assembly shorter than 95% or longer than 105% of the original RefSeq assembly was omitted from analysis to reject pairs of assemblies from read sets that may have included a failed sequencing run. Successful assemblies were annotated with PGAP (build 6021) [9] by supplying the original assembly’s genus and species identifiers. Features and pseudogene types were extracted from the resulting gff files. Only BioSample records with exactly two successful re-assemblies were analyzed to avoid cases where the same BioSample may not correspond to the same genome (e.g., evolve and resequence experiments).

### Simulated sequencing of an *E. coli* genome followed by assembly

To investigate spurious pseudogenes under controlled conditions, we generated simulated sequencing reads starting from the NCBI’s reference *E. coli K12 MG1655* genome (GCF_000005845.2) reannotated with PGAP. Replicate batches (*n* = 3 per condition) of short reads were simulated using the ART (version MountRainier-2016-06-05) [17] simulator under a wide range of coverage depths and qualities. Long reads were simulated with PBSIM3 (v3.0.0) [18] with default quality distributions at fixed 20-fold coverage. All assemblies were generated with Unicycler and annotated with PGAP. Coding sequences were grouped between the assembly and reference genomes with Clusterize using a similarity cutoff of at least 90% nucleotide identity. Spurious pseudogenes were categorized as pseudogenes in the reassembly that were clustered with genes in the reference genome. Only a single pair of nearest neighbors were retained in each cluster with the objective of excluding potentially paralogous coding sequences. The number of spurious pseudogenes due to internal stops or frameshifts were normalized by the total number of coding sequences in each assembly. We fit binomial models to predict this value from simulated coverage and quality. The binomial model assumes spurious pseudogenes are sampled from a finite pool of possible coding features at a rate that is dependent on coverage and/or quality. Model fitting was performed with the *glm* function in R (v4.2.1). Whole genome alignment was performed using DECIPHER to verify that no reassemblies exactly recapitulated the original assembly.

### Reassembly using subsampled reads from very high coverage genomes

Available SRA runs were subset to single Illumina sequencing runs with more than 1000-fold coverage and less than 10,000-fold coverage. Where available, we included single Oxford-Nanopore or Pacific Biosciences sequencing runs with more than 80-fold coverage and less than 1000-fold coverage. Matching BioSamples were further randomly subsampled to at most 100 representatives of each Illumina sequencing model or combination of short and long read sequencing models. Read sets were reassembled at ~1000-fold coverage with Unicycler to obtain a point of reference that was assumed to be the correct assembly. Reads were randomly subsampled in triplicate to target coverages of 5, 10, 25, 50, 100, 250, and 500-fold for short reads, as well as 20-fold coverage for long reads when available. Subsampled reads were reassembled with Unicycler, and contigs shorter than 200 base pairs were dropped before annotation with PGAP.

Successfully reassembled read sets (BioSamples) were further subset to those with at least one completed replicate at all target coverages greater than 5-fold, total assembly size between 80% and 120% of the source assembly size, observed coverages between 80% and 120% of the target coverage, and an alignment fraction (i.e., shared gene content) with the source assembly greater than 80%. These filters were imposed to ensure reasonably complete reassemblies were obtained. Deviation in pseudogene counts from the point of reference (1000-fold coverage) assembly and fitting of binomial models were performed in the same manner as for the simulated *E. coli* reads. Since target coverages were inexact, actual coverages calculated by the SAMtools (v1.18) *depth* function were used for model fitting. We used each fitted model to predict the expected deviation in pseudogenes at 50-fold coverage and the coverage required for a single pseudogene difference per Mbp.

### Electronic supplementary material

Below is the link to the electronic supplementary material.

Supplementary Material 1

## Data Availability

The scripts and datasets used in this study are available from GitHub (https://github.com/npcooley/Pseudogenes). Data for reproducing all analyses are provided on Zenodo (accession numbers: 8360505, 8361514, 8356318, 8366931, 8378433, 10621233, 10622276, 10625340).
