# Supplementary material for: Many purported pseudogenes in bacterial genomes are bona fide genes
Source: BMC Genomics. 2024 Apr 15;25:365. doi: 10.1186/s12864-024-10137-0 (PMC11017572; doi:10.1186/s12864-024-10137-0)
Supplement: Supplementary file 1 — Supplementary Material 1 [file 12864_2024_10137_MOESM1_ESM.pdf]

# Supporting materials for:

Many purported pseudogenes in bacterial genomes are bonafide genes

Nicholas P. Cooley, Department of Biomedical Informatics, University of Pittsburgh
Erik S. Wright, Department of Biomedical Informatics, University of Pittsburgh

## Table of Contents

**Figure S1: Mapping disagreements for *E. coli* strain NR 51487** **2**

**Figure S2: Pseudogene distributions within species** **3**

**Figure S3: Fitted model coefficients for assemblies from simulated *E. coli* reads** **4**

**Figure S4: Modeled behavior of assemblies from simulated reads by average quality score** **5**

**Figure S5: Modeled behavior of assemblies from simulated single end reads** **6**

**Figure S6: Average Nucleotide Identity (ANI) correlations with assembly measures** **7**

**Table S1: Pseudogene distributions by submitter choices** **8**

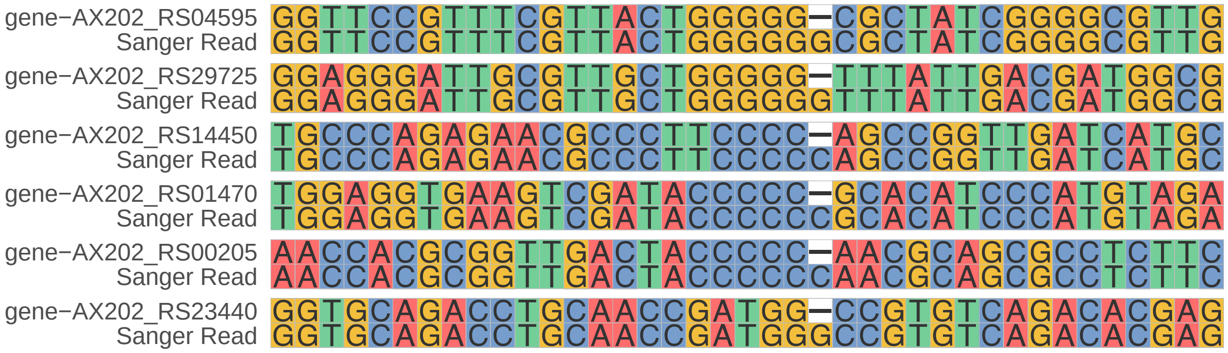


## Figure S1: Mapping disagreements for *E. coli* strain NR 51487

Alignments of Sanger reads implying assembly errors in the deposited assembly for *E. coli* strain NR 51487 (RefSeq accession: GCF_001593565.1). Of ten successfully generated reads mapping to the deposited RefSeq genome, six imply incorrect frameshifts in a gene annotated as a pseudogene.

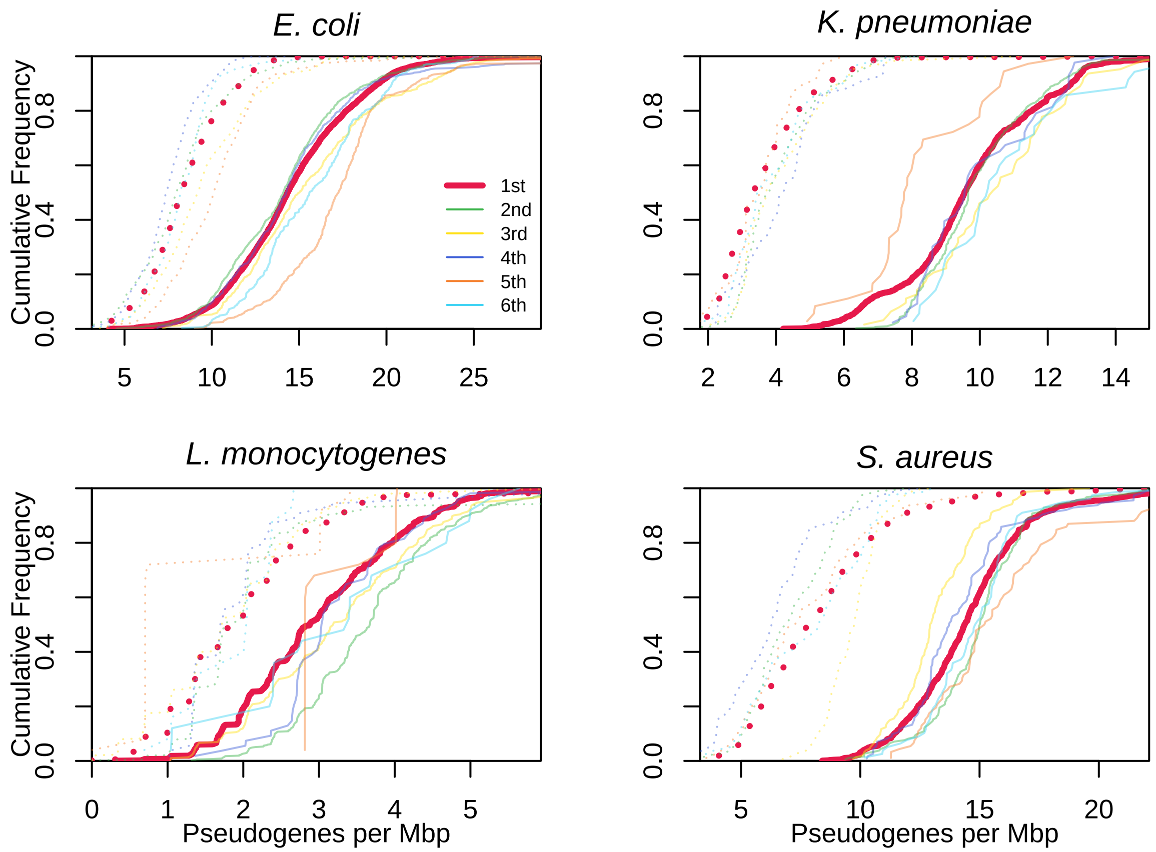


## Figure S2: Pseudogene distributions within species

The top four most common species with captured assembler metadata were split into the top six most common combinations of submitter choices for reported assembler and technology represented by available SRA reads. The most common choice category in every species was a combination of SPAdes and a single SRA run of Illumina reads, and is represented in bold in each plot. Internal stops are represented by dotted lines, and frameshifts are represented by solid lines. The top four species present in this data, in order are *E. coli* (top left), *L. monocytogenes* (bottom left), *K. pneumoniae* (top right), and *P. aeruginosa* (bottom right). Descriptions of comparisons of the minor submitter choice combinations with the major submitter choice combination are present in **Table S1**. Inset legend names refer the rows in the associated supplemental table.

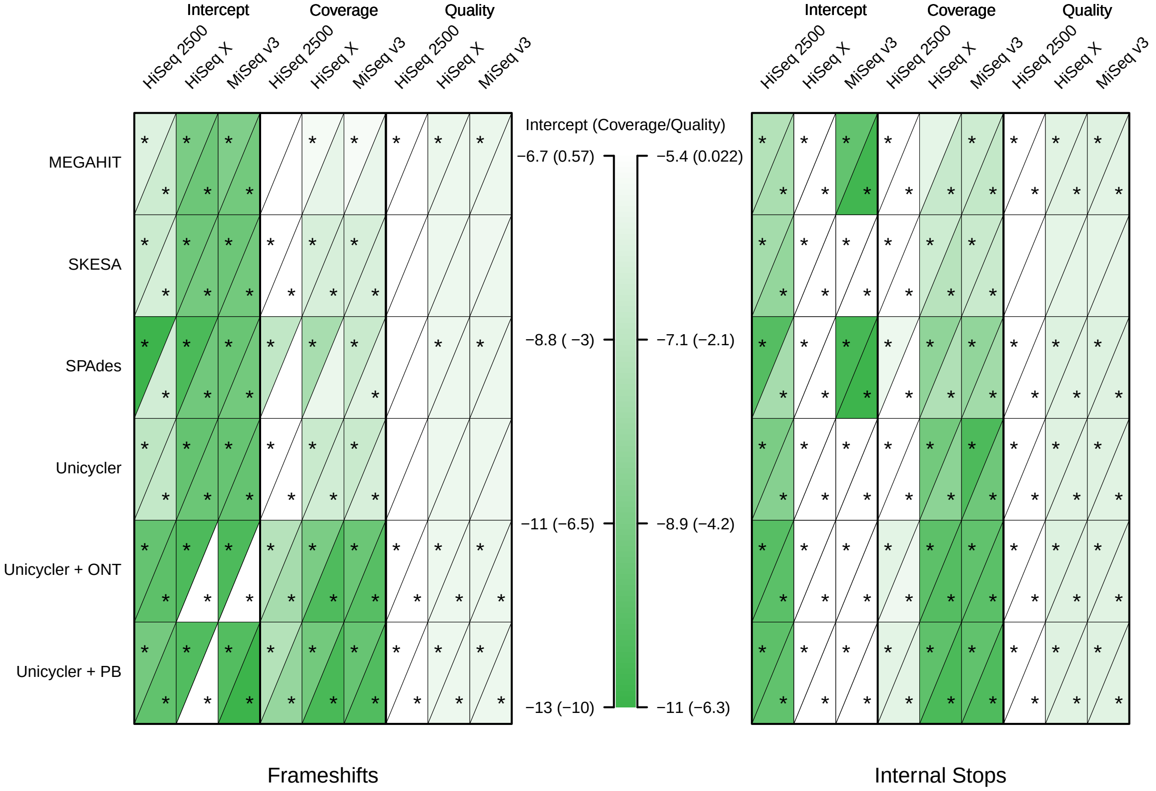


# Figure S3: Fitted model coefficients for assemblies from simulated *E. coli* reads

Coefficients for modeled intercept, coverage, and quality for combinations of simulated Illumina sequencing model and assembler. Each cell is divided diagonally with the upper diagonal representing paired end reads, and the lower diagonal representing single end reads. Coefficients with a Bonferonni corrected p-value $< 0.01$ are appended with an asterisk (*) in the cell bisect. The logistic regression coefficients give the change in log odds of the number of pseudogenes per assembly given a unit increase in the fold coverage or Q-score.

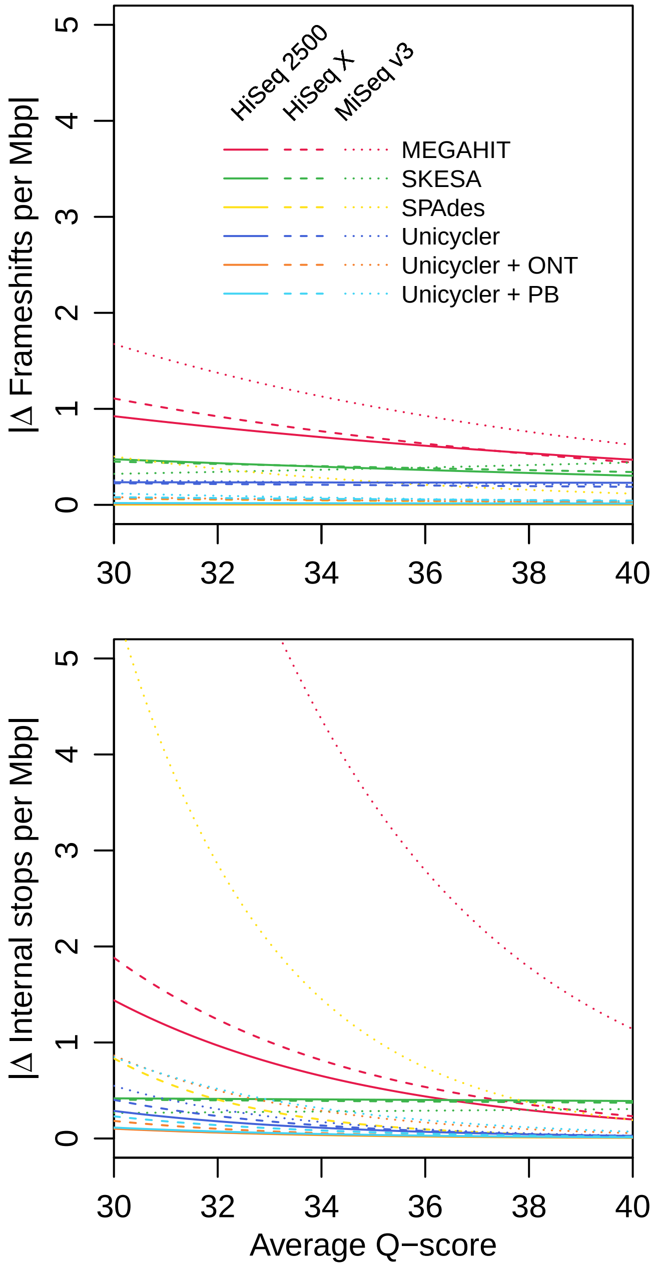


**Figure S4: Modeled behavior of assemblies from simulated reads by average quality score**

Predicted models for the absolute difference in frameshifts (top) and internal stops (bottom) per Mbp from a source genome for assemblies generated from simulated reads as average quality scores vary for an array of sequencing platforms and assemblers. Results present are at a fixed coverage of 50-fold.

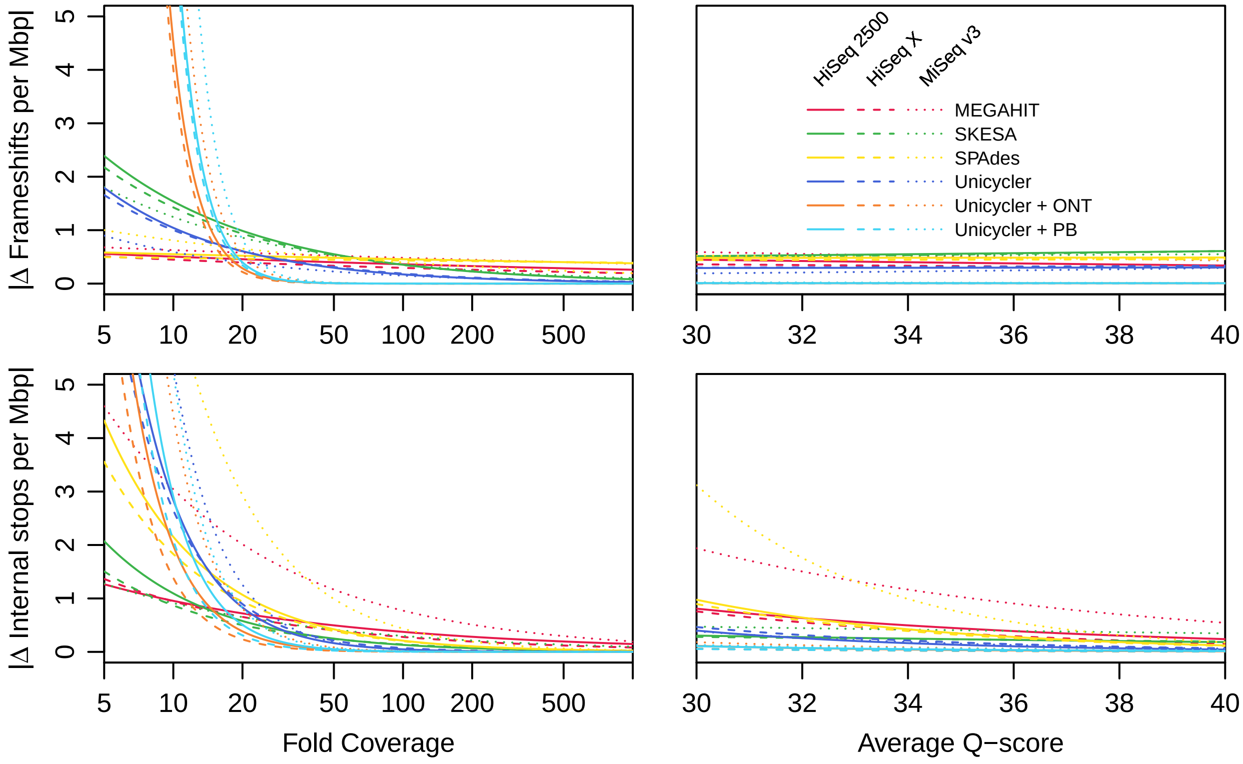


## Figure S5: Modeled behavior of assemblies from simulated single end reads

Predicted models for the absolute difference in frameshifts (top) and internal stops (bottom) per Mbp from a source genome for assemblies generated from simulated single end reads as coverage varies against a fixed quality score of 34 for the short reads (left) and as quality score varies against a fixed coverage of 50-fold for the short reads (right).

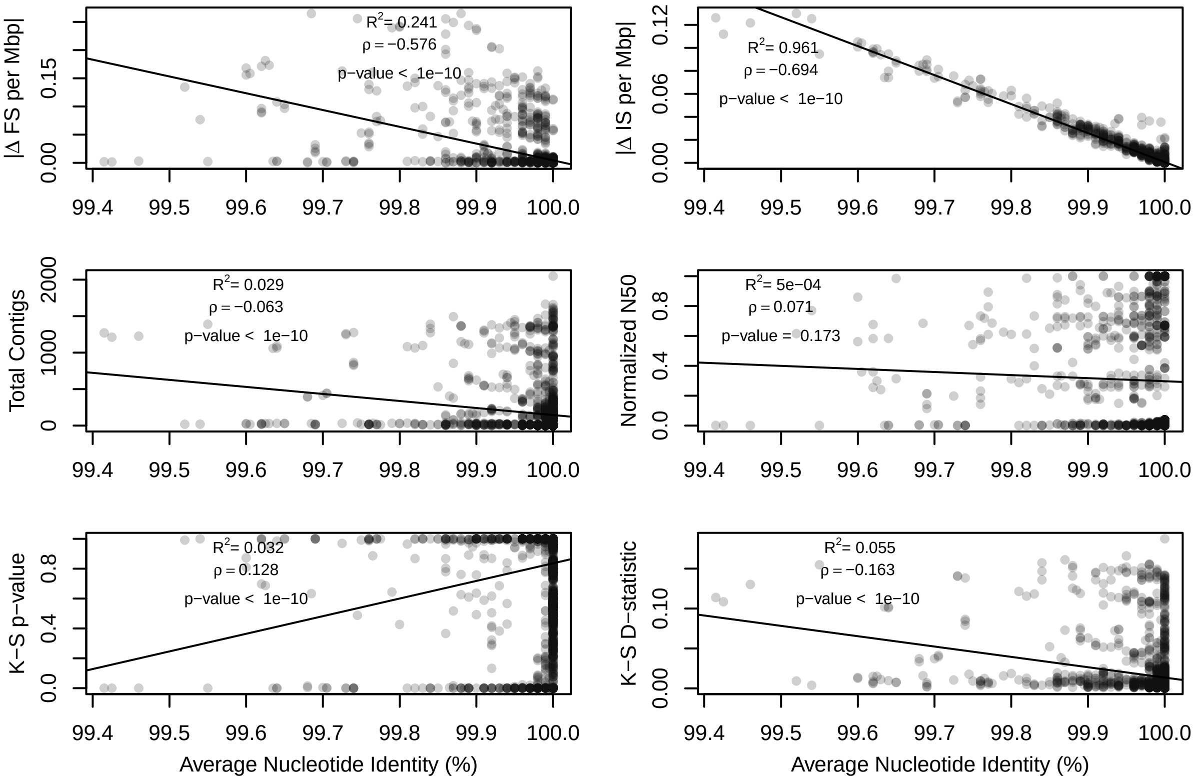


## Figure S6: Average Nucleotide Identity (ANI) correlations with assembly measures

ANI is shown versus extractable statistics about the assemblies generated from simulated *E. coli* reads. ANI versus the absolute difference between the relative frameshifts per Mbp of the generated assembly and the reference assembly (top left). ANI versus the absolute difference between the relative internal stops per Mbp of the generated assembly and the reference assembly (top right). ANI versus the total number of contigs in the generated assembly (middle left). ANI versus the contig N50 normalized to total nucleotides in the generated assembly (middle right). ANI versus the Kolmogorov-Smirnov test p-value when testing the distribution of CDS lengths for the generated assembly against the distribution of CDS lengths for the source assembly (bottom left). ANI versus the Kolmogorov-Smirnov test D-statistic when testing the distribution of CDS lengths for the generated assembly against the distribution of CDS lengths for the source assembly (bottom right). Coefficient of determination, spearman's rho, and p-value of the fitted slope being significant are included in each panel.

| | FS_p_value | IS_p_value | Counts | sub..choice |
|---|---|---|---|---|
| E. coli 2 | 0.0061950 | 0.0002967 | 793 | ILLUMINA 1x + Platanus |
| E. coli 3 | 0.0000613 | 0.0000000 | 474 | ILLUMINA 1x + CLC |
| E. coli 4 | 0.3326202 | 0.0000000 | 465 | ILLUMINA 1x + Abyss |
| E. coli 5 | 0.0000000 | 0.0000000 | 435 | ILLUMINA 1x + Shovill |
| E. coli 6 | 0.0000004 | 0.0003413 | 306 | ILLUMINA 1x + A5 |
| L. monocytogenes 2 | 0.0000000 | 0.0000000 | 570 | ILLUMINA 1x + CLC |
| L. monocytogenes 3 | 0.0005206 | 0.0622754 | 303 | ILLUMINA 1x + Abyss |
| L. monocytogenes 4 | 0.0010448 | 0.0709566 | 54 | ILLUMINA >= 2x + CLC |
| L. monocytogenes 5 | 0.0000142 | 0.0000000 | 25 | ILLUMINA 1x + Skesa |
| L. monocytogenes 6 | 0.3299107 | 0.2725261 | 25 | ILLUMINA 1x + Unicycler |
| K. pneumoniae 2 | 0.0001039 | 0.0000000 | 412 | ILLUMINA 1x + Unicycler |
| K. pneumoniae 3 | 0.0204717 | 0.0005573 | 63 | ILLUMINA ONT + Unicycler |
| K. pneumoniae 4 | 0.3597246 | 0.0015365 | 43 | ILLUMINA 1x + Shovill |
| K. pneumoniae 5 | 0.0000004 | 0.4100879 | 36 | BGISEQ ILLUMINA + SPADES |
| K. pneumoniae 6 | 0.0290836 | 0.1424514 | 35 | ILLUMINA PACBIO + CANU |
| S. aureus 2 | 0.0001104 | 0.0000007 | 184 | ILLUMINA 1x + CLC |
| S. aureus 3 | 0.0000000 | 0.0000000 | 154 | ILLUMINA 1x + MIRA |
| S. aureus 4 | 0.0936508 | 0.0000000 | 113 | ILLUMINA 1x + Abyss |
| S. aureus 5 | 0.0012244 | 0.1724002 | 92 | ILLUMINA ONT + Unicycler |
| S. aureus 6 | 0.1278721 | 0.3722682 | 78 | ILLUMINA 1x + Velvet |

## Table S1: Pseudogene distributions by submitter choices

The dominant submitter combination in all cases was SPAdes and a single set of Illumina reads. The five accompanying plotted minor distributions for each panel in **Fig. S2** were compared against the major distribution for that species with the Kolmogorov-Smirnov test, recording the p-value for both frameshifts (FS) and internal stops (IS), along with the distribution counts.
